# Multimodality Imaging in Contemporary Cardio-Oncology: Beyond Ejection Fraction

**DOI:** 10.3390/diagnostics16213688

**Published:** 2026-11-08

**Authors:** Maria Giuseppina Veglia, Serena Guasti, Giuseppe Romano, Stefano Oliva, Andrea Tedeschi, Maria Laura Canale, Alice Frangione, Jacopo Santagata, Iacopo Fabiani, Francesco Piroli, Francesca Musella, Fabrizio Oliva, Federico Nardi, Massimo Grimaldi

**Affiliations:** 1 U.O.C. Cardiologia “Unità Coronarica e Emodinamica”, Ospedale Madonna delle Grazie, 81740 Matera, Italy; 2 S.C. Cardiologia 4—Diagnostica e Riabilitativa, ASST Grande Ospedale Metropolitano Niguarda, 20162 Milano, Italy; serena.guasti1992@gmail.com; 3 Azienda Ospedaliera Villa Sofia Cervello, UOC Cardiologia Presidio Cervello, 90146 Palermo, Italy; 4 UOSD Cardiologia di Interesse Oncologico, IRCCS Istituto Tumori Giovanni Paolo II, 70124 Bari, Italy; 5 UOC Cardiologia e UTIC, Ospedale Guglielmo da Saliceto di Piacenza, 29121 Piacenza, Italy; andrea.tedeschimd@gmail.com; 6 UOC Cardiologia, Ospedale Versilia, Azienda USL Toscana Nord Ovest, 55041 Toscana, Italy; 7 Cardiology and Arrhythmology Clinic, Polytechnic University of Ancona, 60126 Ancona, Italy; 8 Division of Cardiology, Istituto Nazionale Tumori-IRCCS-Fondazione G. Pascale, 80131 Napoli, Italy; 9 Dipartimento Cardiotoracico, Fondazione Toscana Gabriele Monasterio, 56124 Pisa, Italy; 10 Dipartimento di Cardiologia, Arcispedale Santa Maria Nuova, AUSL Reggio Emilia—IRCCS, 42123 Reggio Emilia, Italy; 11 Department of Clinical Science and Education, Karolinska Institutet, SE-118 83 Stockholm, Sweden; 12 Cardiology Department, Santa Maria delle Grazie Hospital, 80078 Naples, Italy; 13 Unità di Cure Intensive Cardiologiche, Cardiologia 1-Emodinamica, Dipartimento Cardiotoracovascolare “A. De Gasperis”, ASST Grande Ospedale Metropolitano Niguarda, 20162 Milano, Italy; 14 Dipartimento di Cardiologia, Ospedale Santo Spirito, Casale Monferrato, 15033 Alessandria, Italy; 15 U.O.C. Cardiologia-UTIC, Ospedale Miulli, Acquaviva delle Fonti, 70021 Bari, Italy; 16 ANMCO— Associazione Nazionale Medici Cardiologi Ospedalieri, 50121 Firenze, Italy; 17 HCF Fondazione ANMCO per il tuo Cuore ETS, 50121 Firenze, Italy

**Keywords:** cardio-oncology, cancer therapy-related cardiac dysfunction, multimodality imaging, global longitudinal strain, cardiac magnetic resonance, artificial intelligence, cardiotoxicity

## Abstract

**Background**: Cancer therapy-related cardiac dysfunction (CTRCD) may develop before symptoms or a measurable decline in left ventricular ejection fraction (LVEF). Surveillance therefore requires assessment beyond LVEF alone. **Content**: This narrative review examines cardiotoxic mechanisms, imaging findings, and treatment-specific surveillance. Transthoracic echocardiography with LVEF and global longitudinal strain remains the first-line approach. Myocardial work may provide additional information, but oncology-specific action thresholds remain unvalidated. Cardiac magnetic resonance complements echocardiography when measurements are inconclusive or tissue characterization is required. Mapping abnormalities are nonspecific and do not independently establish treatment-related injury. Nuclear imaging addresses selected diagnostic questions; fibroblast activation protein inhibitor PET remains investigational. Artificial intelligence may support image analysis and data integration, although evidence from general cardiovascular populations requires oncology-specific validation. An algorithm and surveillance matrix link clinical findings, treatment, baseline risk, imaging, and biomarkers. **Conclusions**: A tiered approach organizes surveillance around specific clinical questions. Earlier detection should be distinguished from demonstrated clinical benefit: evidence that imaging-guided strategies improve long-term cardiovascular outcomes remains limited. Prospective studies are needed to validate emerging markers, AI applications, and the clinical and economic effects of integrated surveillance pathways.

## 1. Introduction

Over the past twenty years, major progress in cancer therapies has substantially increased survival rates across many malignancies, resulting in a growing population of long-term survivors [1]. This trend has been accompanied by a rise in cardiovascular disease and mortality, now a significant competing risk in oncology patients [2]. The connection between cancer and cardiovascular disease is intricate and bidirectional, influenced by shared risk factors, systemic effects of cancer, and, importantly, the cardiotoxic side effects of treatments. As the field advances, cardio-oncology has emerged to focus not only on minimizing toxicity but also on preserving cardiovascular health during cancer treatment [3]. Traditionally, attention has been on identifying overt cardiotoxicity, mainly symptomatic heart failure (HF) or reduced left ventricular ejection fraction (LVEF), particularly in patients receiving anthracyclines [4]. LVEF may remain normal during early myocardial injury and therefore miss subclinical dysfunction; conversely, an LVEF decline does not necessarily indicate irreversible damage. Newer therapies such as targeted treatments, immune checkpoint inhibitors (ICIs), and multi-drug combinations have expanded the range of cardiovascular side effects to include myocarditis, arrhythmias, vascular complications, and metabolic disturbances, often developing before symptoms or functional decline appear [5]. This evolution highlights the principal limitation of conventional surveillance: although LVEF remains the cornerstone of cardiac monitoring, its dependence on loading conditions, measurement variability, and delayed decline make it an insensitive marker of early cancer therapy-related cardiac dysfunction (CTRCD) [6,7,8]. CTRCD is defined, according to the 2022 European Society of Cardiology (ESC) guidelines, as HF or asymptomatic left ventricular dysfunction attributable to cancer therapy [3]. Asymptomatic CTRCD is graded as mild (LVEF ≥ 50% with a new relative decline in global longitudinal strain [GLS] >15% from baseline and/or a new rise in cardiac biomarkers), moderate (a new LVEF reduction by ≥10 percentage points to 40–49%, or by <10 percentage points to 40–49% with either a new relative GLS decline >15% or a new biomarker rise), or severe (a new LVEF reduction to <40%), whereas symptomatic CTRCD is graded from mild to very severe according to symptom severity and the intensity of HF treatment required [3]. CTRCD is increasingly recognized as a dynamic continuum extending from early molecular injury to overt ventricular dysfunction. Biomarkers and sensitive imaging parameters such as GLS enable detection of myocardial injury before a measurable decline in LVEF occurs [9,10]. Multimodality imaging, increasingly complemented by artificial intelligence (AI), has become a central component of contemporary cardio-oncology. Nevertheless, transthoracic echocardiography with LVEF and GLS assessment remains the guideline-endorsed core of cardiac surveillance [3], whereas the other modalities are complementary and should be used to address specific clinical questions.

**Scheme 1:**
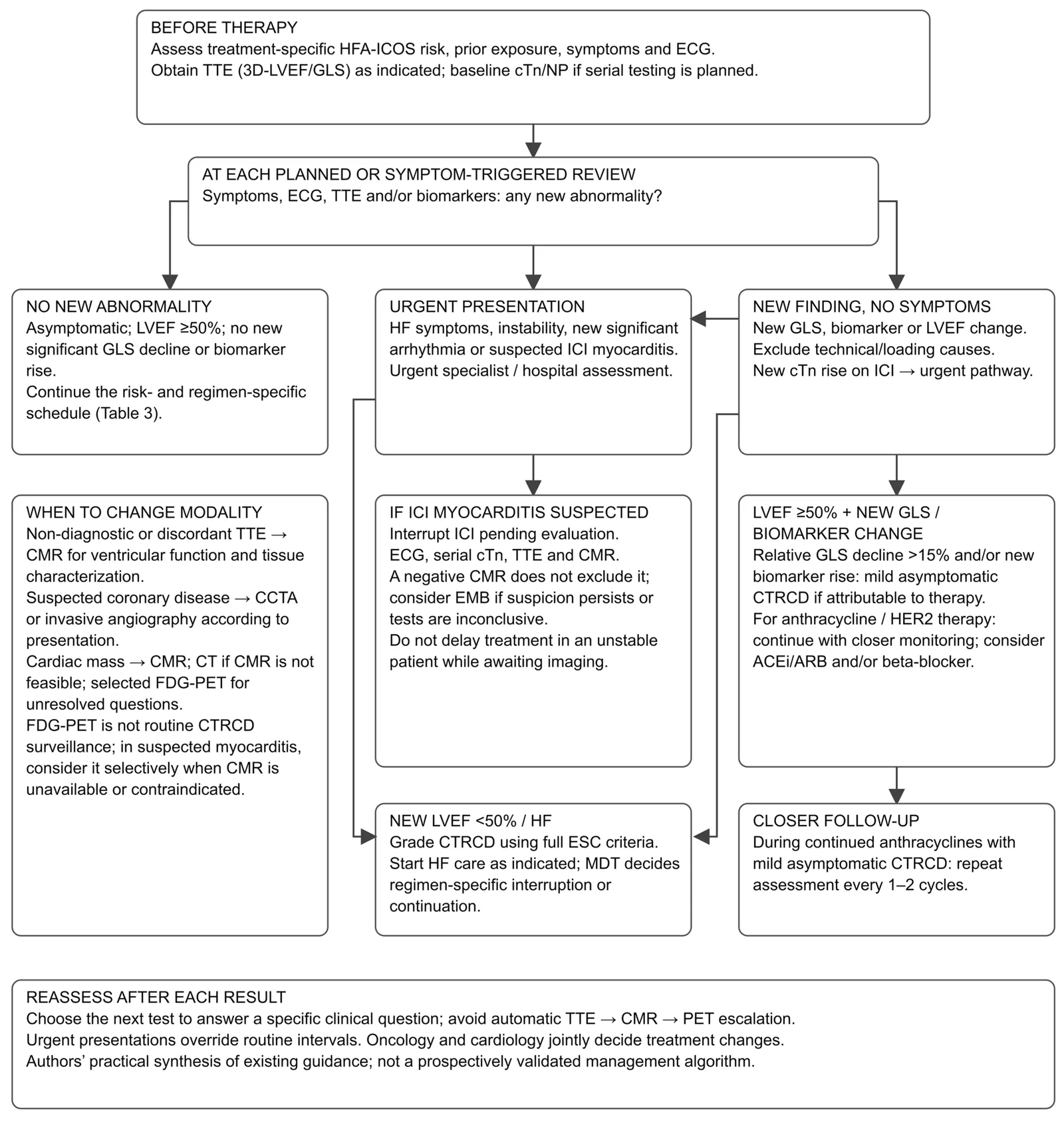
**Operational clinical algorithm for imaging and biomarker surveillance during cancer therapy.** The proposed algorithm integrates symptoms, imaging findings, and cardiac biomarkers to guide further investigation, multidisciplinary assessment, and treatment decisions. For LVEF 40–49%, moderate asymptomatic CTRCD requires a new decline of ≥10 percentage points, or a smaller decline together with a new GLS decline of >15% and/or biomarker rise; a new LVEF < 40% defines severe asymptomatic CTRCD. Baseline LV dysfunction alone is not new CTRCD. GLS changes are relative to baseline. Biomarker thresholds and routine intervals are specified in Section 7.2. ACEi, angiotensin-converting enzyme inhibitor; ARB, angiotensin receptor blocker; CTRCD, cancer therapy-related cardiac dysfunction; EMB, endomyocardial biopsy; MDT, multidisciplinary team; other abbreviations as in the text. HFA-ICOS, Heart Failure Association–International Cardio-Oncology Society; cTn, cardiac troponin; NPs, natriuretic peptides; LVEF, left ventricle ejection fraction; CMR, cardiac magnetic resonance; CCTA, coronary computed tomography angiography.

Building on current guidelines and scientific statements [3,5], this review adds a pragmatic, tiered framework linking baseline risk, imaging modality, and surveillance intensity (Section 7.2), a synthesis of therapy-specific imaging phenotypes (Section 3 and Section 6), and an explicit distinction between guideline-endorsed, emerging, and investigational applications, including AI and opportunistic cardiovascular imaging. An operational branching algorithm (Scheme 1, Section 4.4) and a risk- and treatment-specific imaging/biomarker surveillance matrix translate this synthesis into clinical decision points.

## 2. Methods

This narrative, state-of-the-art review provides a clinically oriented synthesis of multimodality imaging for the detection, characterization, and surveillance of cancer therapy-related cardiac dysfunction. PubMed/MEDLINE and Embase were searched for studies published from January 2015 to July 2026. The search combined terms related to cardio-oncology, cardiotoxicity, multimodality imaging, and artificial intelligence. Earlier landmark studies and additional key references were identified by manually screening relevant reference lists and major society documents. Studies were selected on the basis of their relevance to imaging in cardio-oncology, methodological quality, recency, and clinical applicability. Priority was given to contemporary guidelines and consensus statements, randomized trials when available, large prospective cohorts, and recent meta-analyses. Given the limited number of trials evaluating imaging-guided strategies, observational evidence and expert consensus also informed the synthesis. Diagnostic thresholds and surveillance intervals are linked to the relevant guidelines or primary studies, with established recommendations distinguished from emerging and investigational applications. No formal quality assessment or risk-of-bias scoring was performed.

## 3. Mechanisms and Temporal Evolution of Cancer Therapy-Related Cardiac Dysfunction

### 3.1. Mechanisms of Myocardial Injury Induced by Chemotherapy, Targeted Therapies, and Immunotherapy

#### 3.1.1. Chemotherapy

Anthracyclines, particularly doxorubicin (DOX), remain the prototype of CTRCD. Despite their proven clinical effectiveness, long-term DOX use is limited by dose-dependent cardiotoxicity, which can lead to anthracycline-induced cardiomyopathy (AIC). In a prospective cohort of 2625 patients receiving anthracycline-containing regimens, cardiotoxicity (defined as an LVEF decline > 10 percentage points to <50%) occurred in 9% of cases, 98% of which occurred within the first year after treatment completion [11]. Risk increases continuously with cumulative dose, without a truly safe threshold: the incidence of HF reaches approximately 5% at a cumulative doxorubicin dose of 400 mg/m^2^ and 26% at 550 mg/m^2^ [4,12], and the 2022 ESC guidelines consider cumulative doses ≥ 250 mg/m^2^ a high-risk feature [3]. Strategies to reduce this risk are discussed in Section 3.2. Anthracycline cardiotoxicity arises from a complex interplay of molecular mechanisms involving oxidative stress, mitochondrial dysfunction, DNA damage, and cardiomyocyte death. Through iron-dependent redox cycling, DOX generates excessive reactive oxygen species (ROS), leading to oxidative injury of DNA, proteins, and lipids, impaired mitochondrial function, and progressive myocardial remodeling [13,14]. Mitochondrial accumulation of DOX further disrupts oxidative phosphorylation, depletes ATP production, and activates apoptotic pathways, ultimately contributing to irreversible cardiomyocyte loss [15].

Another key mechanism involves inhibition of topoisomerase IIβ (Top2β), an enzyme involved in DNA repair and transcriptional regulation in cardiomyocytes. Stabilization of the Top2β-DNA cleavage complex by DOX induces double-strand DNA breaks, mitochondrial dysfunction, and maladaptive transcriptional responses that further amplify myocardial injury [16].

Increasing evidence also suggests that susceptibility to AIC is influenced by pharmacogenomic variability. Genetic variants affecting drug transport, metabolism, oxidative stress, calcium handling, DNA repair, and autophagy may partly explain the marked interindividual variability in cardiotoxic risk observed among patients receiving anthracyclines [17].

#### 3.1.2. Targeted Agents

Unlike anthracyclines, therapies targeting the human epidermal growth factor receptor 2 (HER2), including trastuzumab and pertuzumab, typically induce cardiac dysfunction that is largely reversible, although persistent injury and incomplete recovery may occur. Their cardiotoxicity results from inhibition of the HER2/human epidermal growth factor receptor 4 (HER4)-neuregulin-1 signaling pathway, which plays a pivotal role in cardiomyocyte survival, repair, and adaptation to stress [18]. Under normal conditions, HER2 activation stimulates phosphoinositide 3-kinase (PI3K)/protein kinase B (Akt) and extracellular signal-regulated kinase 1/2 (ERK1/2) pathways, protecting the heart from oxidative and mechanical stress. Inhibition of HER2 disrupts these adaptive mechanisms, resulting in contractile dysfunction, particularly in patients with reduced cardiac reserve. Unlike anthracycline-induced injury, this process is predominantly functional rather than structural, and cardiac function frequently recovers after treatment discontinuation [19].

In addition to impairing pro-survival signaling, HER2 inhibition increases mitochondrial susceptibility by reducing the cardiomyocyte’s ability to counteract ROS and maintain mitochondrial homeostasis. Emerging preclinical evidence also suggests that trastuzumab enhances susceptibility to ferroptosis, an iron-dependent form of regulated cell death, through alterations in lipid metabolism, downregulation of glutathione peroxidase 4 (GPX4), and increased mitochondrial lipid peroxidation [20]. Although cardiac dysfunction associated with anti-HER2 therapies is generally reversible, prior exposure to anthracyclines substantially increases the risk of persistent myocardial injury, supporting the concept of a “two-hit” model of cardiotoxicity resulting from the sequential effects of anthracyclines and HER2-targeted therapies [21].

Over the past decade, newer anti-HER2 agents and trastuzumab-based antibody–drug conjugates have become prominent in treating both advanced and early breast cancer, each with unique cardiac toxicity profiles [19,22]. In a meta-analysis of 21 phase 3 trials (9538 patients) in ERBB2-positive advanced breast cancer, the pooled incidence of LVEF decrease was lowest with trastuzumab emtansine (0.94%) and similar with trastuzumab deruxtecan (4.2%), trastuzumab plus chemotherapy (4.9%), and trastuzumab plus pertuzumab plus chemotherapy (5.5%). However, these comparisons were largely indirect, heterogeneity was substantial for some regimens (I^2^ up to 81%), and LVEF decrease was not uniformly defined across trials [22].

#### 3.1.3. Immunotherapy

Immunotherapy (IT) with ICIs restores antitumor immunity by blocking inhibitory T-cell signaling pathways. Currently approved ICI targets are cytotoxic T-lymphocyte-associated protein 4 (CTLA-4), programmed cell death protein 1 (PD-1), its ligand PD-L1, and lymphocyte-activation gene 3 (LAG-3), whereas additional targets, such as T-cell immunoglobulin and ITIM domain (TIGIT), remain investigational. These molecules regulate distinct phases of the immune response: CTLA-4 primarily acts during T-cell priming in lymphoid tissues, whereas PD-1/PD-L1 mainly modulates effector T-cell activity within the tumor microenvironment. Owing to their substantial survival benefit across multiple malignancies, ICIs have become a cornerstone of modern cancer therapy [23,24,25].

Although the toxicity profile of ICIs is generally favorable, their use is associated with specific immune-mediated side effects (immune-related adverse events—irAEs) that can affect every organ and system, both acutely and chronically, and may present with unusual clinical features [26,27].

Cardiovascular irAEs include myocarditis, pericarditis, pericardial effusion, arrhythmias, and vasculitis, with myocarditis being the most severe [27]. Reported incidence varies with data source and case definition: in a French nationwide cohort of 172,363 ICI-treated adults, myocarditis occurred in 0.3–0.6% of patients depending on the ICD-10-based definition, with a higher risk of myotoxicity with combination ICI therapy [28]. Observational studies also suggest late cardiovascular toxicities with prolonged ICI exposure, including accelerated atherosclerotic disease, which may remain clinically unrecognized for extended periods; incidence estimates vary widely according to endpoint definition and follow-up duration [24,29].

The molecular basis of immunotherapy myocarditis remains unclear, but recent preclinical evidence points to an abnormal immune response targeting myocytes. Translational studies have identified clonally expanded T cells specific for α-myosin [30] and pathogenic cardiac macrophages [31] as potential drivers of myocarditis, although these mechanisms have not yet been fully delineated in humans [27].

Beyond myocarditis, immune checkpoint pathways such as CTLA-4 and PD-1/PD-L1 contribute to vascular immune homeostasis and protection against atherosclerosis. Their pharmacological inhibition may accelerate plaque development and destabilization through enhanced CD4+ T-cell-driven vascular inflammation, with additional contributions from cytotoxic T cells in advanced lesions. These processes promote cholesterol accumulation, endothelial dysfunction, and smooth muscle cell remodeling, thereby increasing long-term cardiovascular risk during ICI therapy [25,29,32].

### 3.2. Temporal Progression and Reversibility of Cancer Therapy-Related Myocardial Injury

Patients with cancer may already have increased cardiovascular risk or subclinical cardiac abnormalities before starting treatment. This baseline vulnerability should be distinguished from CTRCD, which refers to cardiac dysfunction attributable to anticancer therapy [3]. Systemic inflammation, oxidative stress, neurohormonal activation, autonomic dysfunction, metabolic abnormalities, and clonal hematopoiesis contribute to cardiovascular vulnerability in patients with cancer. This pre-existing vulnerability may increase susceptibility to myocardial injury once anticancer therapy starts [33].

Among anticancer therapies, anthracyclines provide the best-characterized model of the temporal evolution of CTRCD, in which the molecular mechanisms described above progressively translate into subclinical myocardial injury, ventricular dysfunction, and ultimately overt HF if left untreated. Clinically, anthracycline cardiotoxicity has traditionally been classified as acute, early-onset chronic progressive, and late-onset toxicity. Acute toxicity, occurring during or shortly after drug administration, is uncommon, generally stress-mediated, and usually reversible following treatment discontinuation. In contrast, early-onset toxicity, the most frequent presentation, is characterized by an asymptomatic decline in LVEF, whereas late-onset toxicity is associated with progressive ventricular remodeling, dilated cardiomyopathy, and symptomatic HF [11,34]. This time-based classification describes the timing of onset and is complementary to the 2022 ESC framework, which grades CTRCD by severity irrespective of timing [3]; moreover, prospective data show that most cases occur within the first year, suggesting that early and late forms largely represent a continuum of the same injury [11].

Myocardial injury can precede detectable functional impairment. Troponin release during anthracycline treatment may identify cardiomyocyte injury before a measurable LVEF decline [4]. Progression depends on cumulative exposure, baseline vulnerability, and the capacity for repair. Risk rises continuously with cumulative dose; a clinically reliable threshold below which myocardial injury cannot occur has not been established [35,36,37]. Cumulative doxorubicin exposure ≥ 250 mg/m^2^ is used by the ESC to identify increased treatment-related risk and guide surveillance; it is not a biological threshold separating safe from unsafe exposure [3,4].

Although anthracyclines and HER2-targeted therapies are traditionally regarded as paradigmatic examples of irreversible and reversible cardiotoxicity, respectively, this distinction is increasingly recognized as an oversimplification. Anthracycline-induced cardiotoxicity is generally dose-dependent and characterized by permanent structural cardiomyocyte injury, whereas HER2-targeted therapies predominantly cause a dose-independent, functional impairment that frequently recovers after treatment discontinuation. Nevertheless, prior anthracycline exposure, advanced age, pre-existing cardiovascular disease, hypertension, and diabetes may reduce myocardial reserve, leading to delayed or incomplete recovery after HER2 inhibition and challenging the concept of universally reversible cardiotoxicity [38].

Sensitive imaging parameters, particularly GLS, together with cardiac biomarkers, may identify subclinical myocardial damage before irreversible remodeling develops, although definitive evidence demonstrating improved long-term clinical outcomes is still limited. Preventive strategies such as limiting cumulative anthracycline exposure, using liposomal formulations or continuous-infusion schedules, and administering dexrazoxane have been shown to reduce cardiotoxic risk [3,4]. However, despite decades of investigation, pharmacological approaches for the primary prevention of CTRCD have produced conflicting results, and no strategy has consistently demonstrated clinically meaningful benefit across unselected patient populations [39,40]. By contrast, targeted cardioprotection in patients with imaging- or biomarker-defined subclinical injury has a stronger rationale: the 2022 ESC guidelines recommend considering angiotensin-converting enzyme inhibitors or angiotensin receptor blockers and/or beta-blockers in mild asymptomatic CTRCD [3]; in the SUCCOUR trial, fewer patients developed CTRCD at 1 year with GLS-guided cardioprotection, but the primary endpoint of LVEF change did not differ significantly, and the trial was not powered for clinical outcomes [10]; and in SUCCOUR-MRI, cardioprotection attenuated LVEF decline in patients with GLS-defined subclinical dysfunction [41]. These findings support imaging surveillance aimed at detecting injury before overt ventricular dysfunction develops.

### 3.3. Other Cancer Therapies

Beyond these paradigmatic classes, other anticancer therapies cause cardiovascular toxicity through distinct mechanisms, with corresponding imaging phenotypes [3,42]. VEGF-pathway inhibitors reduce nitric oxide bioavailability and cause endothelial dysfunction and capillary rarefaction, leading mainly to hypertension and, less frequently, left ventricular (LV) dysfunction; serial transthoracic echocardiography (TTE) with GLS, interpreted in light of blood pressure changes, is the main imaging tool [3,42]. Proteasome inhibitors, particularly carfilzomib, are associated with HF, hypertension, and acute coronary syndromes, likely through impaired cardiomyocyte proteostasis and endothelial dysfunction; baseline TTE and natriuretic peptides are recommended [3,42]. Fluoropyrimidines predominantly cause coronary vasospasm and endothelial injury, presenting as chest pain, acute coronary syndrome, or Takotsubo-like syndrome, for which exclusion of obstructive coronary disease by invasive coronary angiography or coronary computed tomography angiography (CCTA) and cardiac magnetic resonance (CMR) for differential diagnosis are the key imaging steps [3]. B-Raf proto-oncogene serine/threonine kinase (BRAF) and mitogen-activated protein kinase kinase (MEK) inhibitors may cause usually reversible LV dysfunction and hypertension by interfering with RAF–MEK–ERK pro-survival signaling [3,42]. Finally, thoracic radiotherapy induces endothelial and microvascular injury, inflammation, and progressive fibrosis, resulting, after a latency of years to decades, in coronary, valvular, pericardial, and myocardial disease, for which TTE, CCTA, and CMR provide complementary information [3,43]. Imaging-based surveillance for these therapies is discussed in Section 6. A brief paragraph on long-term surveillance in survivors of childhood and adolescent cancer is included in Section 6.3, while a detailed discussion of sex- and age-related differences remains beyond the scope of this review.

## 4. Multimodal Imaging for Early Detection of Subclinical Cardiac Dysfunction

### 4.1. Advanced Echocardiography: Myocardial Deformation and Myocardial Work

Transthoracic echocardiography (TTE) remains the cornerstone of cardiovascular surveillance during cancer therapy owing to its wide availability, safety, and suitability for serial assessments. Although LVEF remains the reference parameter for defining CTRCD, its limited sensitivity for detecting early myocardial injury and its dependence on loading conditions restrict its value for identifying subclinical cardiotoxicity [3,6,7]. Moreover, conventional two-dimensional (2D) LVEF relies on geometric assumptions and is susceptible to image foreshortening and interobserver variability, reducing its reproducibility [44]. To overcome these limitations, three-dimensional (3D) echocardiography is recommended whenever feasible, as it eliminates geometric assumptions and provides more accurate and reproducible measurements of ventricular volumes and LVEF, with performance approaching that of cardiac magnetic resonance (CMR) [3,44]. Nevertheless, even 3D-LVEF remains influenced by dynamic changes in loading conditions, which commonly occur during cancer treatment because of intravenous fluid administration, pain, or sympathetic activation [3].

Myocardial deformation imaging by speckle-tracking echocardiography (STE) has become the cornerstone of early cardiotoxicity detection. GLS is the most extensively validated parameter for identifying subclinical myocardial dysfunction and reflects longitudinal shortening of the subendocardial fibers, which are particularly vulnerable to treatment-related injury [45,46]. Reductions in GLS may precede changes in LVEF and are associated with subsequent CTRCD, although variation in thresholds and risk of bias limit certainty [45]. In the 2022 ESC Guidelines, mild asymptomatic CTRCD (LVEF ≥ 50%) is defined by a new relative decline in GLS of >15% from baseline and/or a new rise in cardiac biomarkers, whereas moderate and severe CTRCD are graded mainly by LVEF thresholds [3]. In the SUCCOUR trial, the primary endpoint of change in LVEF did not differ significantly between groups, although fewer patients in the GLS-guided group developed CTRCD; the study was not powered to demonstrate an improvement in heart failure or other clinical outcomes [10]. GLS therefore supports early detection, while its impact on hard outcomes remains unproven. Because GLS measurements remain affected by inter-vendor variability, serial follow-up should be performed using the same ultrasound platform and analysis software whenever possible [3].

Although most studies have focused on left ventricular function, increasing evidence indicates that the right ventricle (RV) is also susceptible to cancer therapy-related injury, particularly in patients receiving anthracyclines or those with pre-existing pulmonary hypertension or lung disease [3]. RV longitudinal strain (RV-LS), especially of the free wall, may identify early RV dysfunction before conventional parameters, although the data are limited [46,47]. Validated oncology-specific cut-offs are lacking; as an approximate reference, RV free-wall strain values > −20% are considered abnormal in the general population [44] and should therefore be interpreted with caution, preferably as a change from baseline and in combination with other RV parameters.

A recognized limitation of GLS is its dependence on afterload, as acute blood pressure changes may influence strain values independently of intrinsic myocardial function. Myocardial work (MW) has been developed to overcome this limitation by integrating GLS with non-invasive blood pressure to generate left ventricular pressure-strain loops [48]. In oncology cohorts, small studies have reported associations between MW indices and CTRCD, suggesting a possible incremental value over GLS, although the evidence remains limited [49,50] and requires confirmation in larger prospective oncology studies. Moreover, MW relies on non-invasive blood pressure as a surrogate for left ventricular pressure, which may itself vary during therapy (e.g., with VEGF inhibitors or changes in volume status), and healthy reference ranges have been reported, but oncology-specific diagnostic and treatment thresholds are not standardized [51].

### 4.2. Cardiac Magnetic Resonance for Myocardial Tissue Characterization

CMR complements TTE when ventricular function cannot be assessed reliably or when tissue characterization may clarify the diagnosis. Native T1 and T2 mapping, extracellular volume (ECV), and late gadolinium enhancement (LGE) provide information on myocardial composition and injury patterns. These findings require clinical interpretation and should not be equated with histological diagnosis [52,53,54].

Native T1 and ECV may increase during or shortly after treatment, but the changes are nonspecific [52]. In a prospective multicenter cohort of 136 women receiving sequential anthracyclines and trastuzumab, mean native T1 increased from 1012 to 1035 ms and ECV from 25.2% to 26.8% at peak follow-up values [53]. These modest changes were transient and overlapped between patients with and without CTRCD, limiting their value for routine risk prediction. T1 and ECV may reflect edema, inflammation, fibrosis, or a combination of these processes; study-specific values are not transferable diagnostic thresholds, and their long-term prognostic significance remains uncertain [52,53].

T2 mapping detects myocardial edema and active inflammation. Its clinical relevance has become particularly evident with the widespread use of ICIs, where elevated T2 values support the diagnosis of immune-mediated myocarditis. The updated Lake Louise criteria combine at least one T2-based marker with at least one T1-based marker, the latter including native T1, ECV, or LGE [54]. A normal CMR does not exclude ICI myocarditis, particularly early in its course: LGE, for instance, was present in only about half of the cases in a multicenter series [55,56], so clinical assessment and biomarkers remain essential. CMR may also identify myocardial changes after thoracic radiotherapy, although available cohorts are small and findings are heterogeneous [55].

LGE identifies focal myocardial injury and replacement fibrosis. Its distribution helps distinguish ischemic from nonischemic disease, but a subepicardial or mid-wall pattern is not specific for treatment-related myocarditis or toxic cardiomyopathy [54,57,58]. LGE is often absent after anthracycline treatment, and its absence does not exclude diffuse myocardial injury [53]. Oncology-specific prospective data establishing the prognostic value of individual LGE patterns remain limited.

CMR provides reproducible measurements of ventricular volumes and function and can characterize pericardial inflammation and associated tissue abnormalities. TTE remains the initial examination for effusion and its hemodynamic consequences; CT and CMR provide complementary assessment when thickening, inflammation, or malignant involvement is suspected [55,59].

The wide field of view also enables evaluation of cardiac masses and intracardiac thrombi [55,59].

Gadolinium use should be individualized according to renal function, contrast-agent risk group, and the expected diagnostic benefit [60]. For repeated examinations, the need for contrast should be reassessed rather than assumed; native mapping can answer some questions without additional gadolinium exposure [57,60]. Long acquisition times, arrhythmias, patient tolerance, and access may limit CMR. Implanted ports and devices require verification of MR conditions and consideration of image artifacts, rather than automatic exclusion [3,57,61].

CMR has broadened the evaluation of CTRCD from the assessment of ventricular function alone to comprehensive myocardial tissue characterization, but its impact on patient management and clinical outcomes remains to be fully established [52].

### 4.3. Nuclear and Molecular Imaging Approaches

Nuclear imaging complements structural imaging in selected clinical settings, while several molecular applications remain investigational. Multigated acquisition (MUGA) provides reproducible LVEF measurements but entails ionizing radiation and limited structural information. For ventricular function surveillance, it is a third-line option when TTE and CMR are unavailable or cannot provide a diagnostic assessment [3,62].

Positron Emission Tomography (PET), especially when combined with CT (PET/CT), may help in the diagnosis of inflammatory cardiotoxicity. 18F-fluorodeoxyglucose (18F-FDG) PET/CT is used in selected patients with suspected ICI-related myocarditis, where it can reveal intense, patchy, or widespread myocardial glucose uptake, indicating infiltration by activated T-lymphocytes and inflammatory cells [62]. A major limitation is physiological myocardial glucose uptake: adequate patient preparation, with a high-fat, low-carbohydrate diet and prolonged fasting (with or without intravenous heparin), is essential to suppress it [62]. Even with optimal preparation, myocardial FDG uptake is not specific for cardiotoxicity, and its interpretation therefore requires integration with clinical, biomarker, and CMR findings [62]. Sensitivity for ICI-associated myocarditis is imperfect; a negative PET does not exclude myocarditis, and its incremental value over CMR remains uncertain [55,62].

Beyond inflammation, molecular imaging can explore specific pathways of cardiomyocyte injury. 123I-metaiodobenzylguanidine (123I-MIBG) scintigraphy assesses the cardiac sympathetic nervous system, and a reduced heart-to-mediastinum ratio has been reported as a marker of anthracycline-induced damage in some small studies preceding the decline in LVEF [62,63]. This evidence is limited, and the technique is available only in a few centers.

Fibroblast activation protein inhibitor (FAPI) PET targets activated fibroblasts but remains investigational in CTRCD; validated thresholds and evidence supporting surveillance or treatment decisions are lacking [62,63].

Repeated nuclear examinations add radiation exposure to an often substantial diagnostic imaging burden. This is particularly relevant in younger patients and long-term survivors. Exposure varies with tracer, administered activity, camera technology, and the CT component; repeated testing should therefore be justified by a specific clinical question, optimized to limit dose, and replaced by TTE or CMR when these can provide equivalent information [3,62].

Clinically, nuclear imaging mainly serves as a “problem-solving” tool for complex cases or high-risk patients. With advances in personalized oncology, molecular imaging within multimodality approaches may expand and eventually help tailor cardioprotective strategies to each patient’s biological response to therapy [3,62].

### 4.4. Incremental Value of Integrated Multimodality Imaging

Multimodality imaging combines complementary information to address specific diagnostic and management questions (Scheme 1). The choice of test depends on the suspected mechanism of injury, baseline risk, and the reliability of preceding measurements (Table 1) [3,55,64].

In routine practice, a tiered rather than universal strategy is supported by guidelines, consensus statements, and observational data, rather than by randomized comparative evidence [3,5,61].

TTE with GLS is the first-line modality for baseline assessment and serial surveillance when indicated by treatment and cardiovascular risk. CMR is useful when TTE is non-diagnostic or discordant, or when tissue characterization is required. In suspected ICI myocarditis, FDG-PET may be considered selectively when CMR is unavailable or contraindicated; it is not a routine next step after CMR and should not delay treatment or biopsy when clinically indicated [3,62].

Multimodality imaging is also central in cardiac masses, metastases, and thrombi, which are frequent clinical occurrences in patients with cancer. TTE (or CT) usually detects the lesion; CMR tissue characterization helps differentiate thrombus, benign tumors, and metastatic involvement; and FDG-PET/CT contributes metabolic information and extracardiac staging. Integrated approaches have been reported to improve diagnostic accuracy over single modalities [61,65,66], in line with a tiered, not universal, strategy. In a prospective single-center study of 142 patients with cardiac masses (54% malignant, including metastases) with histology or resolution after anticoagulation for thrombi as the reference, CMR had the highest standalone accuracy for malignancy. In inconclusive CMR cases, a positive FDG-PET discriminated malignancy accurately, and the combination of CMR and FDG-PET reached an AUC of 0.97, although comparisons between combined strategies were underpowered [66].

These comparative data concern cardiac masses, not CTRCD surveillance. In practice, non-diagnostic TTE prompts CMR; new GLS or biomarker abnormalities prompt clinical reassessment and closer surveillance; suspected ICI myocarditis requires urgent evaluation, with PET reserved for selected unresolved cases [3,62,66].

Integrating imaging findings with clinical characteristics and circulating biomarkers may help distinguish transient functional abnormalities from true myocardial injury. The expected benefit is to reduce unnecessary interruption of potentially life-saving anticancer therapies while enabling timely initiation of cardioprotective treatment in patients at highest cardiovascular risk [3,65,67]; this is a plausible rationale supported mainly by observational data, and prospective evidence of improved oncological or cardiovascular outcomes is still lacking.

## 5. Artificial Intelligence in Cardio-Oncology Imaging

### 5.1. Automated Image Analysis for Early Detection and Risk Stratification

Artificial intelligence (AI) is increasingly being investigated as a means of improving the reproducibility, efficiency, and scalability of cardiovascular imaging. In cardio-oncology, these capabilities are potentially relevant to serial assessment before, during, and after cancer treatment, when small changes in cardiac function may influence clinical decisions (Table 2). Most studies of AI in imaging, however, have been conducted in general cardiology cohorts rather than in patients receiving potentially cardiotoxic therapies. Table 2 summarizes the clinical applications and potential impact of AI in cardio-oncology.

The strongest evidence for AI in echocardiography concerns measurement automation and reduced analysis time. In a blinded randomized workflow trial involving 3495 echocardiographic studies, the proportion requiring a change of more than 5 percentage points between the initial LVEF estimate and final cardiologist assessment was 16.8% with AI and 27.2% with sonographer assessment [68]. Other studies have evaluated multitask interpretation and single-frame LVEF estimation [69,70]. These results concern general echocardiography populations, not serial CTRCD surveillance. Changes in image quality and loading conditions during cancer treatment may affect performance, and oncology-specific diagnostics and clinical benefit remain unproven.

AI-enhanced CMR has been investigated for diagnosis, estimation of diastolic filling pressure, and scar analysis [71,72]. These general-cardiology applications provide technical evidence but have not established accurate detection of treatment-related myocardial injury in cancer populations.

In serial oncology examinations, automated segmentation and quantitative analysis may reduce analysis time, but changes in acquisition and physiology still require expert review.

Virtual native enhancement (VNE) generates contrast-free images resembling LGE. A prospective two-center study evaluated myocardial infarction scar assessment rather than CTRCD [73]. Across the full image set, pooled diagnostic accuracy was 81.1% for VNE and 92.3% for LGE; in the subset judged suitable for contrast-free assessment, accuracy was 93.7% and 93.9%, respectively. This dependence on image quality and case selection limits extrapolation. VNE remains investigational for treatment-related myocardial injury and is not an established substitute for contrast-enhanced CMR in cardio-oncology.

### 5.2. AI-Enabled Longitudinal Monitoring and Outcome Prediction

AI may support longitudinal risk assessment by combining imaging, ECG, biomarker, and clinical data [74]. However, models developed in general cardiovascular populations have not established reliable prediction of CTRCD, myocarditis, arrhythmias, or HF during cancer treatment.

Automated CMR scar analysis has been associated with arrhythmic events in a general-cardiology registry [72]. For single-lead AI-ECG prediction of first HF hospitalization, reported C statistics were 0.723 in the Yale cohort, 0.736 in UK Biobank, and 0.828 in ELSA-Brasil [75]. The latter two cohorts had only 46 and 31 HF events, respectively, resulting in imprecise estimates. These were not dedicated oncology cohorts. Cardio-oncology-specific calibration, clinically useful thresholds, and the benefit of acting on model predictions have yet to be established.

AI-assisted coronary CT analysis may support vascular risk assessment, including after radiotherapy or treatments associated with vascular toxicity. Current evidence mainly comes from broader cardiovascular cohorts and methodological studies; prediction of treatment-related vascular events remains a proposed application [76,77].

ACC guidance addresses the recognition and management of cardiovascular adverse effects of targeted therapies and ICIs [78]. It does not validate AI monitoring of these complications. Suspected myocarditis or another serious adverse event requires established urgent diagnostic pathways.

### 5.3. Limitations and Future Perspectives

The principal evidence gap concerns the clinical benefit of technically promising AI models in cardio-oncology. Many models have been developed retrospectively, in single-center or narrowly defined populations, without prospective evaluation of their effects on diagnosis, treatment decisions, or patient outcomes. Generalizability across cancer types, treatment regimens, age groups, comorbidities, imaging vendors, acquisition protocols, and healthcare settings therefore remains uncertain. External validation in representative cardio-oncology cohorts, followed by prospective studies and implementation evaluations, should be considered a prerequisite for routine use.

Dataset shift is particularly relevant in oncology: image quality and cardiac physiology may vary during treatment, patients may undergo repeated examinations using different equipment, and the prevalence and spectrum of disease can differ from those in the development cohort. Model evaluation should report discrimination and calibration, clinically meaningful operating thresholds, missing-data handling, subgroup performance, and failure modes. Where applicable, studies should assess whether the model adds value beyond established clinical variables and guideline-based imaging rather than reporting model performance in isolation [77,79,80,81].

Explainability methods, including saliency or activation maps, may help users inspect which image regions contribute to an algorithm’s output, but visual explanations do not by themselves prove that a model is correct or clinically safe. Explainable AI should be combined with external validation, transparent reporting, and assessment of potential bias. Regulatory requirements for software as a medical device, post-deployment monitoring, cybersecurity, and change control should also be considered when implementing models in clinical workflows [79,80,81].

Automation bias is another practical concern: clinicians may rely excessively on seemingly precise AI outputs, particularly when time is limited or image quality is suboptimal. In cardio-oncology, the consequences could include false reassurance, unnecessary interruption of cancer treatment, or delayed investigation of a serious adverse event. A human-in-the-loop model is therefore essential. AI outputs should be reviewed in the context of symptoms, treatment exposure, biomarkers, prior imaging, and established clinical guidance; uncertainty or poor-quality input should trigger verification rather than automatic action [77,79,80,81].

**Figure 1:**
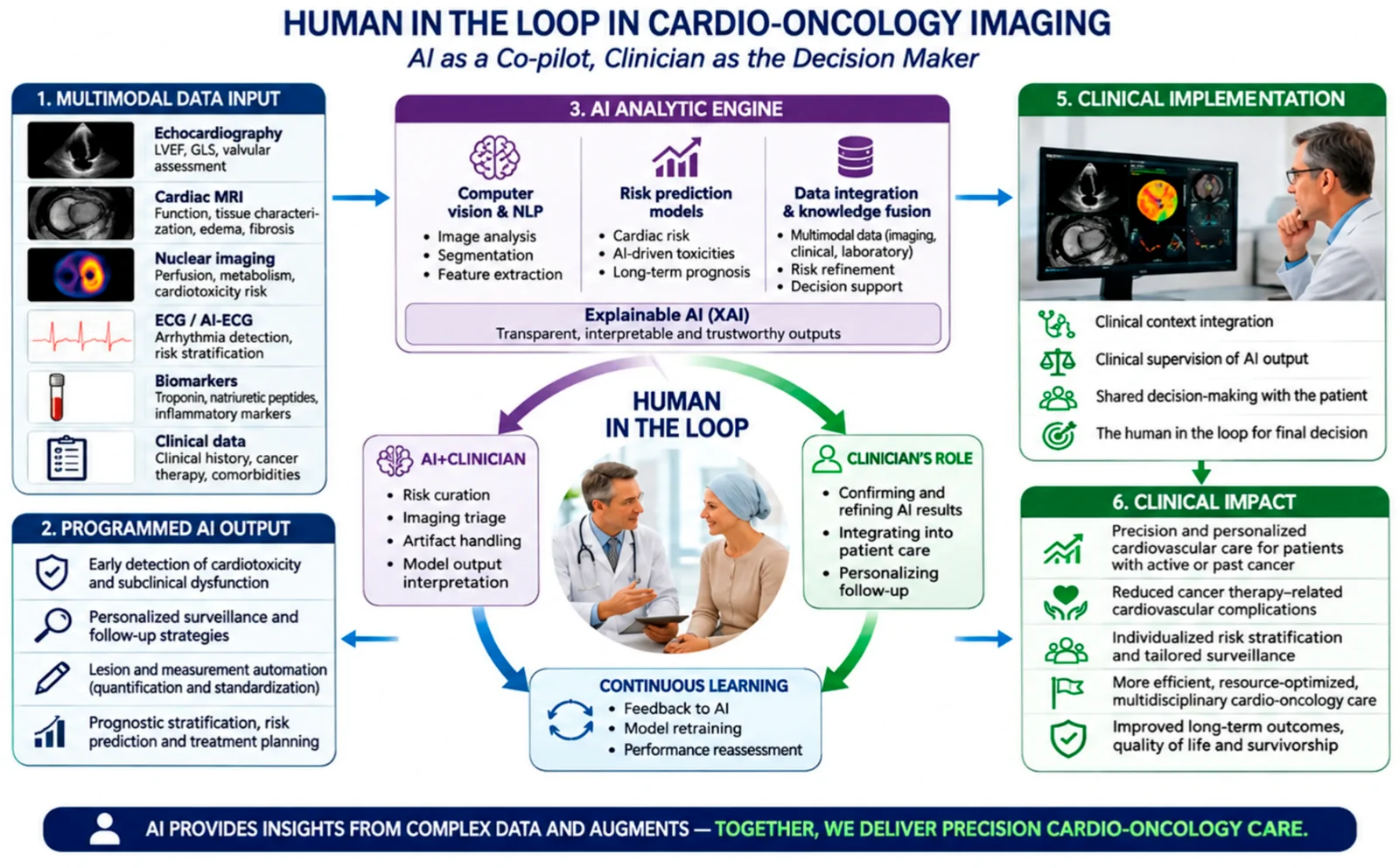
Artificial intelligence-enhanced imaging workflow across the cardio-oncology continuum. Potential roles of artificial intelligence in cardio-oncology imaging. Conceptual framework illustrating how AI-assisted image analysis and data integration may support clinician-led assessment and decision-making. The framework is not a validated prediction model. Proposed benefits, including improved clinical outcomes, safety, and quality of life, remain to be demonstrated in cardio-oncology.

AI supports image analysis and data integration; its value for oncology treatment decisions requires external validation, calibration, and prospective demonstration of clinical utility [74]. Figure 1 illustrates the potential roles of AI in cardio-oncology imaging.

## 6. Imaging-Guided Long-Term Surveillance Across the Cardio-Oncology Continuum

Surveillance during treatment and survivorship should reflect the timing and spectrum of treatment-related cardiovascular injury [42,82].

The myocardial, vascular, inflammatory, and hemodynamic effects of individual treatments are summarized in Section 3. Their differing time courses justify treatment-specific surveillance rather than a single schedule [3,42].

### 6.1. Monitoring Patients Without Detectable Cardiac Injury

For patients without initial signs of cardiac injury who are scheduled to receive potentially cardiotoxic cancer treatments, imaging monitoring should focus on detecting early, subtle abnormalities before clear CTRCD manifests. Baseline imaging should be obtained before treatment when indicated by the regimen and cardiovascular risk. The 2022 ESC guidelines recommend baseline TTE for all patients receiving anthracyclines or HER2-targeted therapy; for other regimens, baseline assessment follows therapy-specific recommendations and cardiovascular risk. A uniform three-month pretreatment window should therefore not be applied to all regimens. Subsequent surveillance should reflect treatment exposure, baseline risk, and interim findings [3]. TTE remains the cornerstone of serial surveillance. For LVEF assessment, 3D echocardiography is preferred; the biplane Simpson method is used when 3D imaging is unavailable [44]. Asymptomatic severe CTRCD is defined by a new LVEF < 40%; moderate CTRCD comprises a new LVEF reduction of ≥10 percentage points to 40–49%, or a smaller reduction to 40–49% accompanied by a relative GLS decline > 15% or a new cardiac biomarker rise. Mild asymptomatic CTRCD requires LVEF ≥ 50% with a new relative GLS decline >15% and/or a new cardiac biomarker rise [3].

Among patients with preserved LVEF, GLS is more sensitive than LVEF for detecting early left ventricular dysfunction. A relative reduction in GLS of >15% from baseline is the ESC threshold for a significant change [3]. This relative reduction refers to the magnitude (absolute value) of GLS: [(|GLS baseline| − |GLS follow-up|)/|GLS baseline|] × 100. Absolute GLS values more negative than −18% are a practical reference rather than a universal normal limit: interpretation depends on age, sex, loading conditions, and vendor/software-specific reference ranges. Serial comparisons should preferably use the same acquisition system and analysis software and prioritize change from the individual baseline [44,46,64]. This is particularly relevant in patients receiving anthracyclines or sequential anthracycline/HER2-targeted therapy, in whom GLS abnormalities may precede a decline in LVEF by weeks or months [10]. In selected high-risk patients, additional abnormalities in RV function (tricuspid annular plane systolic excursion (TAPSE), RV systolic tissue velocity (S′), or RV free-wall strain) and diastolic indices may further support the suspicion of cardiotoxicity, although these parameters are less specific and lack validated cut-offs [47]. Myocardial work indices such as global work index (GWI), global constructive work (GCW), global wasted work (GWW), and global work efficiency (GWE) might add value in borderline cases, particularly when GLS is affected by afterload changes, but their routine use is limited due to lack of standardization [51].

CMR is the preferred secondary test when TTE findings are inconclusive or difficult to reproduce. Increased T2 may reflect edema, whereas increased native T1 and ECV may reflect edema or other interstitial changes; these findings do not independently establish treatment-related injury [53]. Reduced LV mass together with ECV changes may provide additional evidence of remodeling, interpreted alongside ventricular function and the clinical context [83].

For serial ventricular function surveillance, the 2022 ESC guidelines prioritize TTE, followed by CMR when echocardiography is non-diagnostic; MUGA is a third-line option when both are unavailable [3]. Cardiac CT is not a routine modality in this surveillance pathway, although its ventricular measurements have been validated against CMR [84]. This statement concerns function surveillance only: CT retains established indications for coronary assessment and cardiac mass evaluation, including as an alternative when CMR is not feasible [3,59,61].

### 6.2. Imaging Follow-Up in Subclinical Cardiotoxicity

Once subclinical cardiotoxicity is detected, the goal of imaging follow-up is to assess whether the abnormality remains limited to myocardial deformation or progresses toward more severe ventricular dysfunction or symptomatic HF. As an interpretative framework proposed by the authors, rather than a guideline-defined classification or validated prognostic model, follow-up imaging can distinguish three descriptive patterns: stability, where strain impairment persists without worsening; reversibility, indicated by normalization on subsequent assessment, potentially following cardioprotective treatment or oncologic treatment modification; and progression, shown by worsening GLS, declining LVEF, or CMR signs of active myocardial injury and remodeling.

Serial TTE assesses whether a confirmed GLS abnormality persists, resolves, or accompanies a decline in LVEF [46]. RV, diastolic, or left atrial abnormalities may indicate more extensive involvement, although their incremental clinical value and oncology-specific thresholds remain less established [47,64]. Table 1 summarizes routine surveillance before established dysfunction; new abnormalities require closer, individualized follow-up under Scheme 1 [3].

The primary source for regimen-specific surveillance is the 2022 ESC guideline [3]. During HER2-targeted treatment, TTE is recommended every 3 months in the neoadjuvant/adjuvant setting and during the first year of metastatic treatment. In asymptomatic low-risk patients receiving neoadjuvant/adjuvant therapy with a normal assessment after 3 months, the interval may be extended to 4 months; in asymptomatic low- or moderate-risk patients with metastatic disease and no cardiovascular toxicity, it can be extended to 6 months after the first year. During carfilzomib treatment, assessment every 3 cycles should be considered at high/very high risk and may be considered at low/moderate risk. With combined BRAF/MEK inhibition, TTE every 4 months during the first year should be considered at high/very high risk. For VEGF inhibitors, TTE every 3 months in the first year should be considered at high/very high risk; an additional TTE at 4 weeks should be considered in selected high- and very high-risk patients, especially when cardiac biomarker surveillance is unavailable. TTE every 4 months during the first year may be considered at moderate risk [3].

For anthracyclines, high/very high-risk patients require TTE every 2 cycles and within 3 months after completion; all adults should undergo assessment within 12 months. In moderate-risk patients, additional TTE should be considered at a cumulative doxorubicin-equivalent dose ≥ 250 mg/m^2^, and may be considered at low risk [3]. For ICIs, baseline ECG and cardiac biomarkers are recommended for all patients, with baseline TTE recommended at high risk. ECG and troponin monitoring should be considered before doses 2, 3, and 4, then every 3 doses if normal; new symptoms, ECG changes, or biomarker abnormalities require prompt TTE and assessment for myocarditis, including CMR when indicated. Routine serial TTE in otherwise asymptomatic patients has no established universal schedule [3].

In asymptomatic patients receiving anthracyclines or HER2-targeted therapy, preserved LVEF (≥50%) with a confirmed relative GLS decline > 15% meets the ESC definition of mild asymptomatic CTRCD. Continuing anticancer treatment with more frequent cardiac monitoring is recommended (Class I, Level C); an angiotensin-converting enzyme inhibitor/angiotensin receptor blocker and/or beta-blocker should be considered for a significant GLS decline (Class IIa, Level B) [3]. These recommendations do not replace urgent evaluation and treatment interruption when ICI-related myocarditis is suspected. During continued anthracycline treatment with mild asymptomatic CTRCD, monitoring every 1–2 cycles is recommended [3]. Scheme 1 separates routine surveillance, mild asymptomatic CTRCD, reduced LVEF/HF, and urgent suspected ICI myocarditis; Section 7.2. provides the corresponding risk- and treatment-specific testing intervals.

CMR should be considered when TTE findings are inconclusive, discordant, or limited by suboptimal image quality. It provides reproducible ventricular measurements and tissue characterization that may reveal abnormalities before overt functional deterioration.

### 6.3. Long-Term Surveillance After Overt Cancer Therapy-Related Cardiac Dysfunction

After overt CTRCD, follow-up should assess sustained recovery, persistent dysfunction, and subsequent deterioration. Multidisciplinary review should consider symptoms, HF status, arrhythmias, cardiovascular risk factors, treatment exposure, and serial imaging [3]. Annual cardiovascular risk assessment, including ECG and natriuretic peptides, is recommended after potentially cardiotoxic drugs or radiation involving the heart; imaging frequency depends on risk and clinical findings [3,85].

TTE remains the first-line examination for long-term ventricular function surveillance [3]. CMR is useful when recovery is incomplete, echocardiographic findings are discordant, or tissue characterization may influence management. Native T1, ECV, and LV mass abnormalities may persist despite LVEF recovery but are not specific for treatment-related injury. Absent LGE does not exclude diffuse myocardial disease [83,86].

Recovery of LVEF does not by itself establish that cardioprotective therapy can safely be withdrawn. In a retrospective analysis of a single-center prospective cohort, Park et al. studied 134 patients with improved CTRCD (LVEF increase ≥ 10 percentage points): Of those, 90 continued cardioprotective therapy, and 44 withdrew it. Withdrawal was associated with a higher two-year composite risk of HF hospitalization or LVEF decline ≥ 10 percentage points (hazard ratio 3.06; 95% confidence interval 1.51–7.73). The subgroup with LVEF < 45% at CTRCD diagnosis had a lower final LVEF after withdrawal than after continuation (48.2 ± 6.9% vs. 53.3 ± 6.4%) [87]. These observational associations are subject to confounding and do not establish a causal effect of withdrawal. They support a cautious, individualized multidisciplinary approach with continued surveillance, particularly after more severe initial dysfunction [85,87].

Survivors of childhood and adolescent cancer require a distinct long-term pathway, particularly after anthracyclines, mitoxantrone, or radiation involving the heart. The ESC recommends lifelong surveillance for high-risk survivors. In asymptomatic adults who survived childhood or adolescent cancer, TTE surveillance should be considered every 2 years at high/very high risk and every 5 years at moderate risk (Class IIa, Level B for both intervals), alongside annual screening for modifiable cardiovascular risk factors. Prior CTRCD or persistent abnormalities require individualized, closer follow-up and coordinated transition from pediatric to adult care [3].

### 6.4. Imaging Markers of Late Cardiotoxicity and Myocardial Remodeling

Late cardiovascular toxicity is the chronic or delayed expression of treatment-related cardiac injury and may become clinically evident months to years after completion of cancer therapy. Its spectrum is broad, ranging from persistent subclinical dysfunction to overt HF, progressive chamber remodeling, valvular disease, coronary artery disease, and pericardial involvement. Accordingly, it should not be viewed simply as a delayed reduction in LVEF but rather as the manifestation of a progressive remodeling process that may evolve silently over time and remain only partially captured by conventional systolic indices [3,42].

Imaging during long-term follow-up should assess persistent or new GLS abnormalities, LV dilatation, diastolic dysfunction, and incomplete reverse remodeling. Left atrial or RV abnormalities may indicate involvement beyond the LV but require interpretation within the broader clinical picture [64].

CMR may identify late myocardial abnormalities, including diffuse interstitial changes. Elevated native T1 and ECV may reflect edema, fibrosis, or other interstitial alterations and are not specific for treatment-related injury. Reduced LV mass in anthracycline-exposed patients has been associated with cardiomyocyte atrophy in a longitudinal CMR study; it should be interpreted with ECV and ventricular function rather than regarded as a specific standalone measure of cellular loss [83]. Absent LGE does not exclude late myocardial damage, which may be diffuse rather than focal. Conversely, ongoing abnormalities in native T1, ECV, or LV mass may reveal a substrate for chronic dysfunction, even if LVEF appears stable [83,86].

This late phenotype is not limited to the myocardium. In long-term survivors, especially after thoracic radiotherapy, imaging should also assess valvular thickening or calcification, pericardial disease, and radiation-associated coronary disease, which may emerge years to decades after exposure [3,67]. In this setting, TTE remains the first-line screening tool for late valvular and pericardial sequelae, whereas cardiac CT is useful when calcific valvular disease, pericardial thickening, or coronary atherosclerotic burden requires more precise anatomical characterization. In selected survivors, CAC scoring and coronary CCTA may complement TTE, particularly after chest irradiation or in the presence of multiple conventional cardiovascular risk factors [3,67]. In asymptomatic late high-risk adult survivors, TTE should be considered starting 5 years after radiation involving the heart and every 5 years thereafter. In asymptomatic very high- and early high-risk adult survivors, TTE should be considered at years 1, 3, and 5 after completion of cardiotoxic cancer therapy, then every 5 years (Class IIa, Level C for both schedules). In asymptomatic survivors exposed to a mean heart dose > 15 Gy, non-invasive coronary screening should be considered starting 5 years after radiation and repeated every 5–10 years (Class IIa, Level C). These intervals guide screening rather than define a minimum latency: new symptoms or signs of valvular, pericardial, or coronary disease warrant earlier investigation [3].

## 7. Clinical Implementation and Future Directions

### 7.1. Personalized Cardioprotective Strategies Guided by Advanced Imaging and AI

Cardioprotection decisions should integrate baseline risk, treatment exposure, symptoms, biomarkers, and confirmed imaging changes [3,88,89]. TTE with GLS remains the first-line tool. In SUCCOUR, 331 anthracycline-treated patients with an additional HF risk factor were randomized to GLS-guided (relative reduction ≥ 12%) or LVEF-guided initiation of cardioprotective therapy [10]. At one year, the primary endpoint of LVEF change did not differ significantly, although CTRCD occurred in 5.8% versus 13.7%. At three years, 255 patients completed follow-up, and neither LVEF change nor the difference in CTRCD incidence was statistically significant [90]. The trial was not powered for hard clinical outcomes. Myocardial work may refine assessment when afterload changes, but treatment thresholds remain unvalidated [91]. CMR is used for specific questions, particularly suspected ICI myocarditis; urgent treatment should not await imaging in an unstable patient [3,86]. AI may improve measurement workflows, whereas individualized oncology risk prediction still requires prospective validation [74,92]. Opportunistic coronary artery calcium assessment on existing CT may inform vascular risk evaluation [89,93].

### 7.2. Balancing Clinical Value, Cost, and Accessibility

A tiered surveillance pathway begins with TTE and uses advanced imaging when the result can answer a defined clinical question [3,5,61,94]. CMR is most useful for non-diagnostic or discordant TTE findings and for suspected inflammatory, ischemic, or mixed myocardial injury [61,85,86,95]. Nuclear imaging is reserved for selected questions; FAPI-PET assessment of fibroblast activation remains investigational [63,96]. Access also depends on scanner availability, expertise, and local resources [5,61,74]. Portable ultrasound, tele-imaging, and remote interpretation may help extend specialist support [5,74,92]. When advanced imaging is unavailable, TTE and serial troponin/NP testing can support triage, with testing frequency tailored to treatment and Heart Failure Association–International Cardio-Oncology Society (HFA-ICOS) risk [3,97,98]. A new biomarker rise requires assessment for alternative causes and may prompt closer imaging or referral. Biomarkers do not substitute for urgent specialist evaluation of suspected myocarditis or other severe toxicity. Table 3 summarizes routine schedules; Scheme 1 addresses new abnormalities. Prospective cost-effectiveness evidence for integrated multimodality surveillance remains insufficient [3,85,88,89,99].

HFA-ICOS tools assign treatment-specific low, moderate, high, or very high baseline risk on the basis of cardiovascular history, risk factors, prior cardiotoxic exposure, and baseline tests; higher risk warrants closer surveillance using the schedules in Section 6.2 [3,97]. ESC biomarker thresholds are cardiac troponin I/T above the assay-specific 99th percentile, B-type natriuretic peptide (BNP) ≥ 35 pg/mL, or N-terminal pro-B-type natriuretic peptide (NT-proBNP) ≥ 125 pg/mL, or a new significant rise beyond biological and analytical variability. Obtain a baseline value if serial monitoring is planned, preferably using the same assay, and interpret changes in the context of renal function and other causes of elevation [3].

### 7.3. Future Perspectives for Cardio-Oncology Imaging

**Figure 2:**
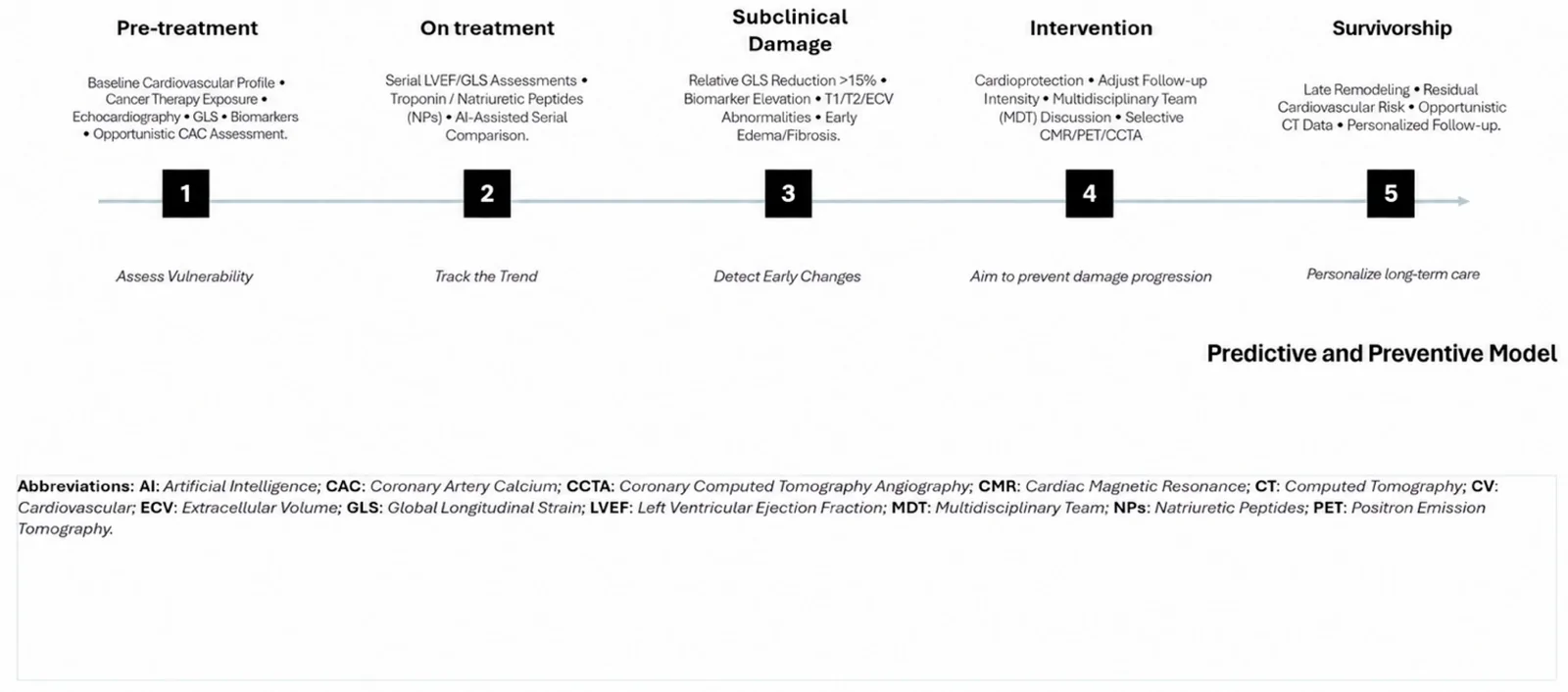
From imaging findings to a predictive cardio-oncology model. Conceptual continuum of imaging-guided surveillance in cardio-oncology, from baseline risk assessment to treatment monitoring, individualized intervention, and survivorship. Abbreviations: AI: Artificial Intelligence; CAC: Coronary Artery Calcium; CCTA: Coronary Computed Tomography Angiography; CMR: Cardiac Magnetic Resonance; CT: Computed Tomography; CV: Cardiovascular; ECV: Extracellular Volume; GLS: Global Longitudinal Strain; LVEF: Left Ventricular Ejection Fraction; MDT: Multidisciplinary Team; NPs: Natriuretic Peptides; PET: Positron Emission Tomography.

Research should determine which imaging and biomarker combinations improve decision-making beyond standardized TTE and baseline clinical risk assessment [61,63,99,100]. Candidate treatment-associated patterns include transient T1/ECV increases during sequential anthracycline/HER2 therapy and edema with nonischemic injury patterns in ICI myocarditis; neither is specific or universally present [53,54,56]. New anticancer therapies may produce myocardial or vascular abnormalities before LVEF changes, but their surveillance pathways require validation [4,89,95,99]. Opportunistic assessment of existing oncological CT may provide CAC information and, with suitable acquisitions, myocardial ECV [93,101]. 2-based breast arterial calcification assessment has shown prognostic associations in a general mammography screening population, rather than a dedicated cardio-oncology cohort [102]. Prospective implementation studies should establish whether these approaches change management, improve outcomes, and justify their additional resource use [74,92]. Figure 2 depicts the conceptual surveillance continuum; it is not a validated predictive model.

### 7.4. Limitations

Several limitations arise from the narrative design of this review. Although the literature search was structured, study selection was not systematic, and no formal quality assessment was performed. The available evidence varies across populations, imaging protocols, and CTRCD definitions. Evidence supporting several imaging applications, including myocardial work, MIBG, FAPI-PET, and most AI tools, is derived from small, single-center, or retrospective studies. Randomized trials demonstrating that imaging-guided strategies improve hard clinical outcomes are lacking. Inter-vendor variability limits the transferability of strain and mapping cut-offs, while the cost and availability of advanced imaging vary widely across healthcare systems. Accordingly, throughout the review, we distinguish guideline-endorsed recommendations from emerging evidence and investigational applications.

## 8. Conclusions

TTE with LVEF and GLS remains the basis of CTRCD surveillance. CMR and nuclear or vascular imaging add value when selected to resolve a specific diagnostic question, with biomarkers and clinical risk informing timing and interpretation [3,5,61,86]. The algorithm and surveillance matrix in this review organize existing guidance into a practical framework; they have not been prospectively validated as an integrated pathway.

Earlier detection does not by itself demonstrate improved outcomes. Evidence that advanced imaging, AI, or integrated surveillance improves long-term cardiovascular outcomes or cost-effectiveness remains limited [5,74,85,92,99]. These benefits require prospective testing before emerging applications can be incorporated into routine cardio-oncology care.

## Figures and Tables

**Table 1:** Overview of multimodality imaging modalities in cardio-oncology.

Imaging Modality	Main Use in Cardio-Oncology	Strengths	Limitations	Clinical Indication or Threshold
**Echocardiography (preferably 3D-LVEF)**	Standard follow-up	Widely available, repeatable	Ejection fraction is a late marker of damage	Baseline and serial surveillance according to treatment and risk [3]
**GLS**	Detection of subclinical myocardial injury	Sensitive, early marker	Dependent on image quality and standardization	Relative reduction > 15% from baseline [3]
**Myocardial work**	Adjunctive functional assessment	Integrates strain with estimated LV pressure	Blood pressure and acquisition dependence; limited oncology validation	No validated oncology-specific diagnostic or treatment threshold [49,50,51]
**CMR**	Assessment of function, inflammation, tissue characterization, and pericardial involvement	Ventricular function; T1/T2 mapping, ECV and LGE for tissue characterization	Access and patient tolerance; mapping abnormalities are nonspecific	Suspected myocarditis or inconclusive echocardiography
**Nuclear Imaging/PET**	Assessment of inflammation, metabolism, and perfusion	Advanced phenotyping	Radiation; preparation-dependent FDG uptake; FAPI remains investigational	Selected cases
**CT/CCTA/CAC**	Opportunistic cardiovascular risk assessment	Information can also be derived from standard oncologic imaging	Radiation exposure and contrast administration when performed as a dedicated examination	High vascular risk or when CT imaging is already available
**AI**	Automation and data integration	Standardization, individualized risk assessment	Validation requirements and potential bias	No validated standalone clinical trigger

Overview of the main applications, strengths, and limitations of imaging techniques and AI tools for detecting and monitoring cardiovascular toxicity during cancer treatment. The table distinguishes established surveillance approaches from complementary techniques and emerging tools whose clinical utility requires further validation. Abbreviations: AI, artificial intelligence; CAC, coronary artery calcium; CCTA, coronary computed tomography angiography; CMR, cardiac magnetic resonance; CT, computed tomography; ECV, extracellular volume; LVEF, left ventricular ejection fraction; GLS, global longitudinal strain; LGE, late gadolinium enhancement; PET, positron emission tomography.

**Table 2:** Clinical applications and potential impact of artificial intelligence in cardio-oncology.

Clinical Domain	AI Application	Potential Clinical Impact
**Echocardiography**	Automated LVEF and GLS quantification	Improved analysis efficiency; oncology-specific performance requires validation
**POCUS**	AI-assisted cardiovascular screening	Potential earlier diagnosis; oncology validation needed
**CMR**	AI-based tissue characterization (investigational in CTRCD)	Potential tissue-marker analysis; clinical utility in CTRCD unproven
**AI-ECG**	Longitudinal monitoring	Potential identification of dysfunction; oncology validation needed
**Coronary imaging**	Plaque characterization	Potential improvement in vascular risk stratification
**Multimodal AI**	Outcome prediction	Potential personalized surveillance; clinical benefit unproven

**Table 3:** Risk- and treatment-specific surveillance integrating imaging and circulating biomarkers.

Treatment and Baseline Risk	Baseline Tests Beyond Clinical Assessment/ECG	Scheduled Imaging	Scheduled Biomarkers
Anthracyclines Low/moderate risk	TTE for all (I B). Obtain cTn/NP if serial monitoring is planned.	Additional TTE at cumulative doxorubicin-equivalent dose ≥ 250 mg/m^2^: moderate risk, IIa C; low risk, IIb C. TTE within 12 months after completion for all (I B).	cTn/NP every 2 cycles and within 3 months after completion: moderate risk or low risk with ≥250 mg/m^2^, IIa C; other low-risk patients, IIb C.
Anthracyclines High/very high risk	TTE and cTn/NP (I B). Cardio-oncology review before treatment.	TTE every 2 cycles and within 3 months after completion (I C); also within the first 12 months (I B).	cTn/NP before every cycle, and 3 and 12 months after completion (I B).
HER2-targeted therapy All risk groups; intensify at high/very high risk	TTE (I B); cTn/NP at high/very high risk (I C). Consider cTn after anthracyclines before HER2 therapy (IIa A).	Early disease: TTE every 3 months and within 12 months after completion (I B). Selected low-risk patients with normal 3-month assessment: every 4 months may be considered (IIb C). Metastatic disease: every 3 months in year 1; selected stable low/moderate-risk patients, every 6 months thereafter. Intensify at high/very high risk.	Early disease, high/very high risk: cTn/NP every 2–3 cycles, then 3 and 12 months after completion (IIa C). Low/moderate risk: baseline, every 3 months and 12 months after completion may be considered (IIb C).
Proteasome inhibitors Especially carfilzomib	TTE in multiple myeloma receiving a proteasome inhibitor (I C). NP at high/very high risk (I C); consider at low/moderate risk (IIa C).	During carfilzomib: TTE every 3 cycles at high/very high risk (IIa B); may be considered at low/moderate risk (IIb C).	NP at baseline and every cycle during the first 6 cycles of carfilzomib or bortezomib (IIa B).
VEGF-pathway inhibitors Risk-dependent surveillance	TTE at high/very high risk (I C); baseline TTE should also be considered at low/moderate risk (IIa C). Baseline NP if used for serial monitoring. Check BP and QTc.	High/very high risk: TTE every 3 months in year 1 (IIa C); consider an additional examination at 4 weeks in selected patients. Moderate risk: every 4 months in year 1 may be considered (IIb C). Lower-risk follow-up is individualized.	High/very high risk: NP at baseline, 4 weeks and every 3 months in year 1 (IIa C). Moderate risk: NP at baseline and every 4 months in year 1 may be considered (IIb C).
Combined BRAF/MEK inhibitors Risk-dependent surveillance	TTE at high/very high risk (I C); may be considered at low/moderate risk (IIb C).	High/very high risk: TTE every 4 months in year 1 (IIa C). Lower-risk testing is individualized and must also follow drug-specific requirements.	No uniform ESC class-specific serial cTn/NP schedule. Test according to symptoms, clinical findings, and individualized risk.
Immune checkpoint inhibitors All patients; TTE at high risk	ECG, cTn and NP; TTE at high risk (e.g., dual ICI, prior cardiovascular disease or cardiotoxic combination).	No universal routine serial TTE interval in asymptomatic recipients. New symptoms, ECG or biomarker abnormalities prompt urgent assessment, TTE and CMR when myocarditis is suspected (Scheme 1).	ECG and cTn before doses 2, 3 and 4; if normal, every 3 doses thereafter should be considered (IIa B). Repeat NP when clinically indicated; do not impose the cTn schedule on NP.
Fluoropyrimidines Especially pre-existing cardiovascular disease	TTE if prior symptomatic cardiovascular disease. Assess coronary risk; selected high-risk patients may warrant coronary screening.	No uniform routine TTE interval. New chest pain requires urgent assessment for coronary vasospasm/ACS; coronary imaging and/or CMR answer the specific differential diagnosis.	No established uniform serial cTn/NP screening schedule. cTn testing is driven by suspected acute myocardial injury.

Surveillance schedules adapted from the 2022 ESC guidelines [3], according to treatment and HFA-ICOS baseline cardiovascular risk [97]. Classes of recommendation (I, IIa, IIb) and levels of evidence (A–C) apply to individual recommendations. TTE includes 3D-LVEF and GLS when feasible. Biomarkers should be measured at baseline before serial monitoring and interpreted in the clinical context; baseline elevation alone does not indicate new CTRCD. New symptoms or abnormal imaging or biomarker findings require reassessment beyond the scheduled intervals, as outlined in Scheme 1. Long-term and radiation-associated surveillance are discussed in Section 6.3 and Section 6.4. ACS, acute coronary syndrome; BRAF, B-Raf proto-oncogene; BP, blood pressure; CMR, cardiovascular magnetic resonance; cTn, cardiac troponin; CTRCD, cancer therapy-related cardiac dysfunction; ECG, electrocardiogram; GLS, global longitudinal strain; HFA-ICOS, Heart Failure Association–International Cardio-Oncology Society; HER2, human epidermal growth factor receptor 2; ICI, immune checkpoint inhibitor; LVEF, left ventricular ejection fraction; MEK, mitogen-activated protein kinase kinase; NP, natriuretic peptide; NPs, natriuretic peptides; QTc, corrected QT interval; TTE, transthoracic echocardiography; VEGF, vascular endothelial growth factor.

## Data Availability

No new data were created or analyzed in this study. Data sharing is not applicable to this article.

## Abbreviations

ACC	American College of Cardiology
ADC	Antibody–drug conjugate
AI	Artificial intelligence
AIC	Anthracycline-induced cardiomyopathy
BNP	B-type natriuretic peptide
BRAF	B-Raf proto-oncogene serine/threonine kinase
CAC	Coronary artery calcium
CCTA	Coronary computed tomography angiography
CMR	Cardiac magnetic resonance
CT	Computed tomography
cTn	Cardiac troponin
CTRCD	Cancer therapy-related cardiac dysfunction
DL	Deep learning
DOX	Doxorubicin
ECG	Electrocardiogram
ECV	Extracellular volume
ESC	European Society of Cardiology
FAPI	Fibroblast activation protein inhibitor
FDG	Fluorodeoxyglucose
GCW	Global constructive work
GLS	Global longitudinal strain
GWE	Global work efficiency
GWI	Global work index
GWW	Global wasted work
HER2	Human epidermal growth factor receptor 2
HF	Heart failure
HFA-ICOS	Heart Failure Association–International Cardio-Oncology Society
ICI	Immune checkpoint inhibitor
irAE	Immune-related adverse event
LGE	Late gadolinium enhancement
LV	Left ventricular
LVEF	Left ventricular ejection fraction
MACE	Major adverse cardiovascular events
MEK	Mitogen-activated protein kinase kinase
MIBG	Metaiodobenzylguanidine
ML	Machine learning
MUGA	Multigated acquisition
MW	Myocardial work
NPs	Natriuretic peptides
NT-proBNP	N-terminal pro-B-type natriuretic peptide
PET	Positron emission tomography
POCUS	Point-of-care ultrasonography
ROI	Region of interest
ROS	Reactive oxygen species
RV	Right ventricular
SaMD	Software as a Medical Device
TAPSE	Tricuspid annular plane systolic excursion
TTE	Transthoracic echocardiography
VEGF	Vascular endothelial growth factor
VNE	Virtual native enhancement
XAI	Explainable artificial intelligence
